# Field-Zone-Associated Transcriptomic and Metabolic Divergence Despite Nearly Identical Observed Chlorophyll in an Agrivoltaic Tea Setting

**DOI:** 10.3390/genes17101221

**Published:** 2026-10-01

**Authors:** Ruifang Wang, Shuai Zhang, Zhengwan Xie, Songsong Shen, Huiping Zhou, Jie Liu

**Affiliations:** 1 School of Tea and Coffee, Puer University, Puer 665000, China; 2 School of Biological and Chemical Science, Puer University, Puer 665000, China

**Keywords:** agrivoltaics, *Camellia sinensis*, shading, transcriptomics, metabolomics, chlorophyll

## Abstract

**Background/Objectives:** Similar leaf chlorophyll in agrivoltaic tea fields may conceal molecular divergence. **Methods:** We reanalysed physiology, RNA sequencing and metabolomics from five field zones (three plant-level replicates each), prioritizing T2 versus T3. Missing plot maps and independent installation replicates limited inference to zone associations. **Results:** T2 and T3 had nearly identical mean total chlorophyll (0.50495 versus 0.50309 mg g^−1^; Welch *p* = 0.920), whereas T2 had higher carotenoids, total sugars, alkaloids, phenols and flavonoids; all five differences had Benjamini–Hochberg false discovery rate (FDR) < 0.05 across 50 physiological tests. Relative to the open-sun control (CK), T2 and T3 had 7 and 6857 differentially expressed genes and 35 and 523 differential unique metabolite profiles, respectively. Omitting one sample from each of T2, T3 and CK yielded 27 combinations, all showing greater transcriptome and metabolome displacement in T3. After adjustment for the leading whole-omics component, exact within-zone permutation supported sample-pattern coupling for glycolysis and porphyrin metabolism (Benjamini–Hochberg FDR = 0.0288 for each). In comparison with four public tea datasets, T1 showed the strongest descriptive rank concordance; T3 showed little concordance. **Conclusions:** Similar chlorophyll means coexist with divergent molecular profiles. These observational results do not establish effects of photovoltaic hardware.

## 1. Introduction

Agrivoltaics co-locates photovoltaic (PV) electricity generation and crop production on the same land, creating an opportunity to improve combined land productivity while reducing direct competition between food and energy production [1,2]. The crop environment below PV structures, however, is not equivalent to a uniform shade cloth. Module geometry, height, spacing, orientation and tracking can alter the mean and temporal variance of photosynthetically active radiation, the direct-to-diffuse light ratio, spectral composition, leaf temperature, humidity and soil water balance. These changes may benefit crops in high-radiation or water-limited settings, but their biological effects are crop- and configuration-dependent [2]. Recent field evidence indicates that PV integration can improve tea productivity and quality under a defined single-axis system [3]. However, molecular variation among tea plants growing in contrasting agrivoltaic field environments remains insufficiently characterized.

Tea (*Camellia sinensis*) is a shade-tolerant perennial crop whose commercial quality is closely linked to light-responsive primary and specialized metabolism. Conventional shade treatments can increase chlorophyll and amino acids, alter catechins and flavonol glycosides, and remodel carotenoid, phenylpropanoid and hormone-related programmes [4,5,6,7,8,9]. These responses depend on cultivar, shade intensity, spectrum, treatment duration and sampled tissue. For example, shading can reverse the yellow leaf phenotype of the light-sensitive cultivar ‘Huangjinya’ through flavonoid and carotenoid regulation [4], while black and coloured nets produce distinguishable transcriptomes even when their nominal shade rates are comparable [6,7]. Metabolomics further shows that shade responses can differ among positions within a tea shoot, consistent with transport and resource redistribution [8]. Consequently, “shade” is not a single biological state.

Leaf chlorophyll is often used as an accessible indicator of shade acclimation. Pigment accumulation, however, is an endpoint shared by several processes and may not report carbon metabolism, photochemical capacity or specialized metabolite allocation. Two field environments could therefore yield similar chlorophyll contents while engaging different transcriptional and metabolic programmes. This possibility is relevant to agrivoltaic systems, although the present archive lacks time-resolved photosynthetic photon flux density (PPFD), spectrum and microclimate records needed to test tracking, intermittency or configuration-specific light mechanisms. Whether similar chlorophyll levels across observed agrivoltaic field environments can coexist with distinct molecular states, and which metabolic pathways distinguish those states, therefore remains unclear.

Here, we reanalysed a five-zone tea dataset comprising physiological assays, RNA sequencing and widely targeted metabolomics. The primary within-dataset comparison was between the author-reported T2 and T3 zones, which had nearly identical observed mean total chlorophyll. We asked four questions. First, did the two zones differ in other biochemical traits and in the magnitude of their molecular departure from the sunlight zone? Second, which photosynthetic, carbon and specialized-metabolism pathways separated them? Third, did transcript and metabolite layers show selective sample-pattern coupling after zone means and dominant global factors were controlled? Fourth, how did the zone-level pathway programmes compare with four public tea BioProjects? The five zone contrasts and the 50-test physiological family were prioritized for reporting in this retrospective reanalysis, rather than prospectively registered; pathway, cross-omics and public-data comparisons were secondary and exploratory. None supports a causal effect of photovoltaic hardware.

## 2. Materials and Methods

### 2.1. Field Zones, Sample Records and Provenance

The supplied dataset contained five author-reported field-zone groups: the unshaded control (CK) and PV-associated zones T1–T4. Each group comprised three plant-level biological replicates collected from three different tea plants, giving 15 samples in total. Leaf material within each plant-level replicate was combined for sample preparation, but the three biological replicates were processed separately and were not pooled with one another. The available records did not identify independent photovoltaic installations, field blocks, plot IDs or the plant identifier linked to each analysis label. CK and T1–T4 are therefore used as zone labels rather than as independently replicated photovoltaic interventions. Each photovoltaic configuration occurred in only one recorded zone, so configuration effects cannot be separated from zone position or other field differences. Project information identifies large-leaf tea with 19-year-old ‘Yunkang 10’ rootstocks and ‘Yunhuang No. 1’ scions. The PV-associated zones were T1, a flexible PV area with 35 mm spacing; T2, a single-axis area; T3, a fixed-axis area; and T4, a flexible PV area with 100 mm spacing. These hardware descriptions could not be checked against an original field map or installation record.

The supplied archive reported mean illuminance values of 1838.4, 427.2, 1096.6, 547.8 and 667.8 lux for CK, T1, T2, T3 and T4, respectively, corresponding to 100%, 23%, 60%, 30% and 36%. The related 2025 field paper reported use of a TES-1334A digital illuminance meter between 13:00 and 14:00, 10 cm above tea plants of comparable growth on the same horizontal line, with five readings per zone and CK as the reference [10]. The present archive does not contain the original reading sheet or a chain-of-custody record linking that protocol and those values to the 15 omics samples, so the published protocol is reported as related programme context rather than assumed to be the measurement method for this sampling batch. The exact measurement date, raw readings, panel height, orientation, tilt, tracking schedule, PPFD, daily light integral, spectrum and microclimate were unavailable. Lux is weighted to human visual sensitivity and is not a substitute for plant-usable radiation. The values are therefore treated as inherited field-zone descriptors, not as evidence of a light dose or photovoltaic configuration mechanism.

Project information states that the photovoltaic panels were installed in April 2024, representative shoots were tagged in early June 2024, and the tagged shoots were monitored for 25 days before sampling. Samples were collected between 13:00 and 14:00 from different plants with the same slope orientation and comparable growth status. The third functional leaf below the apical bud was collected from a current-month shoot and preserved in liquid nitrogen. The related 2025 paper reports the same early June/25-day schedule and third-functional-leaf sampling, with seven plants per zone sampled on a clear day in morning and afternoon periods [10]. All six authors signed a declaration that the present study used a separate sampling batch and reused no biological samples or datasets from the related 2025 paper. The archived row-level chain-of-custody records are incomplete, so this statement cannot be independently verified sample by sample. Analyses retain the recorded zone labels but do not assume independent plot- or installation-level replication.

Physiology source provenance was audited by comparing workbook formulas, literal cells, cached values and report structure across the two supplied packages. Because the measurement workbooks were identical and the reports differed only in administrative fields and branding, the packages were treated as duplicate manifestations of one assay chain. The group membership of the 15 assay codes was retained. The physiology reports contained 15 assay codes (three per zone), but no verified specimen- or plant-ID crosswalk to the 15 omics analysis labels. We therefore cannot establish whether a physiology assay and an omics profile came from the same plant; within-zone row order is not evidence of pairing. Physiology and omics were compared only at the recorded zone level, and no individual-level trait–omics correlations were calculated.

### 2.2. Physiological Measurements and Statistics

The vendor reports contained chlorophyll a, chlorophyll b, total chlorophyll, carotenoids, total flavonoids, total acids, total sugars, total alkaloids, total triterpenoids and total phenols, each with three assay rows per zone. The physiological assays used a SpectraMax Single Mode Reader ABSPlus (Molecular Devices, San Jose, CA, USA), a BSA124S analytical balance (Sartorius, Göttingen, Germany), an HH-6 digital water bath and an H1750R refrigerated high-speed centrifuge (Hunan Xiang Yi Laboratory Instrument Development Co., Ltd., Changsha, China). The archived records do not identify the HH-6 manufacturer or the suppliers of physiological reagents and reference standards.

According to the laboratory protocols, approximately 0.1 g of liquid nitrogen-ground tissue was used for each assay. Chlorophyll a and b were extracted in 10 mL ethanol-acetone (1:1, v/v), quantified from absorbance at 663 and 645 nm, and summed as total chlorophyll; carotenoids were extracted with the same solvent and measured at 440 nm using an extinction coefficient of 250 L g^−1^ cm^−1^. Total flavonoids were measured by the NaNO_2_-Al(NO_3_)_3_ colorimetric reaction at 510 nm with rutin standards, total sugars by acid hydrolysis and the 3,5-dinitrosalicylic acid reaction at 540 nm with glucose standards, total alkaloids by bromocresol green ion-pair extraction at 410 nm with berberine standards, total triterpenoids by the vanillin–perchloric acid reaction at 548 nm with oleanolic acid standards, and total phenols by the Folin–Ciocalteu reaction at 760 nm with gallic acid standards. Total acids were determined by titration with standardized 0.0097 mol L^−1^ NaOH using phenolphthalein as the endpoint indicator.

Zone means, standard deviations (SDs) and raw points were retained. The five primary comparisons were T1–CK, T2–CK, T3–CK, T4–CK and T2–T3. We calculated Welch mean difference tests with 95% confidence intervals (CIs), Hedges’ g and all 20 possible assignments for an exact two-sided 3-versus-3 permutation *p* value. Welch *p* values from the defined family of 50 trait-by-contrast tests (10 traits × five contrasts) were adjusted together by the Benjamini–Hochberg (BH) method [11]. The resulting 50-test false discovery rate (FDR) adjustment was the primary multiplicity analysis, with FDR < 0.05 as the reporting threshold. A second BH adjustment within each trait across its five contrasts was reported as supplementary context. The minimum attainable exact two-sided permutation *p* value with three samples per group is 0.1; this reflects the 20 possible label assignments, not a different significance threshold. No equivalence test was conducted because an equivalence margin was not specified before analysis. Because independent field installations were not documented, *p* values quantify variation among the supplied samples and are treated as exploratory rather than installation-level inference.

### 2.3. RNA Sequencing, Quality Control and Expression Analysis

According to the project-specific vendor report, plant RNA was extracted using an ethanol precipitation and cetyltrimethylammonium bromide (CTAB)-PBIOZOL procedure. RNA concentration was assessed using a Qubit 4.0 fluorometer (Thermo Fisher Scientific, Waltham, MA, USA)/MD plate reader, and RNA integrity was evaluated using a Qsep400 bioanalyzer (BiOptic Inc., New Taipei City, Taiwan). Poly(A)-containing messenger RNA (mRNA) was enriched using Oligo(dT) magnetic beads and fragmented. First-strand complementary DNA (cDNA) was synthesized using random hexamer primers, followed by second-strand cDNA synthesis, purification, end repair, A-tailing, adapter ligation, size selection and polymerase chain reaction (PCR) enrichment to generate the final cDNA libraries. Libraries with 250–350-bp inserts were sequenced as 150 bp paired-end reads on an Illumina sequencing platform (Illumina, San Diego, CA, USA). Adapter trimming and low-quality read filtering were performed using fastp 0.23.2 [12]. Clean reads were aligned to the reference genome assembly GWHAZTZ00000000 using HISAT2 2.2.1 [13], with GWHAZTZ00000000.gff used as the corresponding structural annotation file. Transcript assembly and gene-level quantification were performed using StringTie 2.1.6 [14] and featureCounts 2.0.3 [15], respectively. The exact sequencing instrument model, MD plate-reader model and library-reagent supplier information were not documented in the supplied records.

Independent differential analysis began from the supplied gene-count matrix. Genes with counts per million (CPM) ≥ 1 in at least three samples were retained. Library sizes were normalized by the trimmed mean of M values (TMM), dispersions were estimated robustly, and a no-intercept five-zone design was fitted using edgeR 4.4.0 quasi-likelihood models [16]. The same five primary comparisons used for physiology were tested. An FDR < 0.05 defined model-based statistical significance; an absolute log_2_ fold change ≥ 1 was reported as a secondary effect-size threshold. Because zone-level field replication was not established, these FDR values describe contrasts among the supplied samples and do not estimate uncertainty across independent photovoltaic installations.

For principal component analysis (PCA) and pairwise Pearson sample correlation quality control (QC), TMM-normalized log_2_ CPM values were used. Preranked Kyoto Encyclopedia of Genes and Genomes (KEGG) analysis used signed square-root quasi-likelihood F statistics and fgseaMultilevel (fgsea 1.32.2) with pathways of 10–500 annotated genes, zero lower-bound approximation and BH correction [17]. All independent R analyses used R 4.4.3 and random seed 20260728.

### 2.4. Widely Targeted Metabolomics and Differential Analysis

Samples were vacuum freeze-dried for 63 h using a Scientz-100F lyophilizer (Ningbo Scientz Biotechnology Co., Ltd., Ningbo, China) and ground at 30 Hz for 1.5 min using an MM 400 grinder (Retsch GmbH, Haan, Germany). A 50 mg aliquot was extracted with 1200 μL of prechilled 70% aqueous methanol containing the vendor-reported internal standard at a working concentration of 250 μg/mL, vortexed six times, centrifuged and filtered before ultra-performance liquid chromatography–electrospray ionization–tandem mass spectrometry (UPLC–ESI–MS/MS). The vendor report described preparation of this solution from a 1000 μg/mL stock in 70% aqueous methanol but did not identify the internal standard compound. Chromatography used an ExionLC AD system (SCIEX, Marlborough, MA, USA) and an Agilent SB-C18 column (Agilent Technologies, Santa Clara, CA, USA) with water/acetonitrile containing 0.1% formic acid. The effluent entered an ESI triple-quadrupole–linear ion-trap system operated in multiple-reaction monitoring mode; the exact mass spectrometer model was not supplied. Peaks were integrated in MultiQuant and processed against the in-house Metware Database using secondary spectral and retention information. The vendor workbook classified 802, 493 and 1637 annotation rows as levels 1, 2 and 3, respectively. These are vendor database-match categories, not evidence of confirmation with authentic standards. Three pooled QC injections were present. Among 2931 rows with complete QC triplicates, the median coefficient of variation (CV) was 14.6%, 86.9% had CV ≤ 30% and all had CV < 50%. Acquisition order, sample randomization and a separate signal drift correction procedure were not documented. The archived vendor method documentation lists methanol and acetonitrile from Merck (Darmstadt, Germany); the formic-acid supplier entry could not be independently verified because its recorded catalogue number conflicts with the supplier name.

The raw vendor workbook contained 2932 annotation rows; 2931 had complete values for the 15 study samples and three pooled QC injections and formed the primary matrix. Exact equality across all 18 columns identified redundant annotation rows. One abundance vector was retained for modelling for each of the 2709 independent exact profiles, while all annotation-to-KEGG links were carried to the corresponding profile ID. A profile could therefore enter more than one supported pathway when alternative annotations linked it to different pathways, but it was counted only once within any one pathway. Intensities were transformed as log_2_(x+1) and median-centred within sample. Limma 3.62.1 models used the same design and comparisons as the transcriptome, with empirical Bayes moderation, trend and robust estimation [18]. An uncentred log_2_ sensitivity analysis was conducted for all annotations and independent profiles. Metabolite pathway conclusions apply only to the 425 mapped independent profiles, or 15.7% of the independent metabolome. Full annotation, QC and mapping details are provided in Supplementary Table S22.

### 2.5. Threshold-Independent Response Magnitude

To compare the scale of field-zone-associated change without relying on significance counts, we enumerated all 3^5^ = 243 ways to omit one of three samples independently from each recorded zone. These all-zone combinations were used to rank the four zone–CK responses. Each individual zone–CK contrast has nine unique omission pairs. For the primary T3:T2 comparison, one of the three samples was omitted from each of T2, T3 and CK, yielding 3^3^ = 27 possible omission combinations; omissions from T1 and T4 were irrelevant because they did not change either response. Within every reduced dataset, group means were recomputed for 31,778 TMM log_2_CPM genes and 2709 normalized metabolite profiles. The primary response magnitude was the median absolute group-mean log_2_ difference across all retained features. T3:T2 stability was assessed over these 27 combinations; the 243 all-zone combinations were retained only for four-zone ranking and audit. This index quantifies multivariate displacement and is not an estimate of energetic or fitness cost.

### 2.6. Group-Aware Cross-Omics Permutation for Biologically Selected Pathways

Eight pathways were selected because they represented the pigment, photosynthesis, carbon and specialized metabolism processes central to the study question: glycolysis/gluconeogenesis, photosynthesis, photosynthesis-antenna proteins, carbon fixation in photosynthetic organisms, porphyrin metabolism, carotenoid biosynthesis, phenylpropanoid biosynthesis and flavonoid biosynthesis. No prospectively registered pathway protocol was available; these analyses are therefore described as biologically selected and exploratory rather than prespecified. All metabolite annotations were mapped to exact-profile IDs so that alternative annotations sharing a measured profile did not create artificial independent observations. Within each pathway, transcript and metabolite features were standardized after zone means were removed. The RV coefficient [19], a multivariate measure of similarity between matrices, was the primary statistic for the resulting pair of 15 × 15 sample similarity matrices.

The null distribution was obtained by exact enumeration of all 6^5^ = 7776 permutations of metabolite sample labels within the five recorded zones, preserving zone structure. Seven biologically selected pathways had enough mapped features in both modalities to be tested. Exact upper-tail *p* values were BH-adjusted across these seven pathways. For global factor sensitivity, each modality’s leading whole-omics principal component (PC1) was removed from the within-zone residual matrices before RV coefficients and exact tests were recomputed, and the adjusted *p* values were again BH-corrected across the same seven pathways. Robustness was additionally examined over all 3^5^ leave-one-per-zone combinations. RV coefficients are interpreted as sample-pattern associations, not causal edges.

### 2.7. External Cross-Study Contextualization

On 28 July 2026, the National Center for Biotechnology Information (NCBI) Sequence Read Archive (SRA) was queried through E-utilities using “*C. sinensis*”[Organism] AND (shade OR shading OR light). Returned experiment packages were enriched with study, experiment, run, sample and sample-attribute metadata and summarized at BioProject level. Inclusion and exclusion decisions were fixed before downstream expression direction was examined. Primary qualification required an interpretable lower light, shade or light intensity comparison in tea with biological replication sufficient for within-study inference; unreplicated, confounded and nonspecific retrievals were excluded. Wavelength experiments were retained only on a separate secondary spectral axis. The query returned 23 BioProjects: six primary intensity, one primary sensitivity, one primary provisional, three secondary spectral and 12 excluded. Qualified projects were expanded to project-complete SRA metadata. The complete decision ledger, selected contrasts and source links are reported in Supplementary Table S20. This cross-study contextualization compares generic lower-light programmes; it is not an independent validation of a photovoltaic configuration or mechanism.

GSE97659/PRJNA356134 contained three sunlight-grown and three shaded ‘Huangjinya’ samples [4]. Raw fragment count columns embedded in the processed table were filtered, normalized and fitted with edgeR. CRA004002/PRJCA004626 contained 18 libraries across the CK, 70% black, 95% black, 95% blue, 95% yellow and 95% red net groups [6]; its published abundance count table was fitted across all five shade-versus-CK contrasts. PRJNA1134925 contained triplicate ‘Echa 10’ RNA-seq libraries after 14 days at 100 or 400 μmol m^−2^ s^−1^ [20]. Its paired reads were quantified by kallisto 0.51.1 against the Plant GARDEN *C. sinensis* var. sinensis (CSS) v2 coding sequences, then filtered and fitted with edgeR. GSE142753/PRJNA598211 comprised triplicate control, five-day shade, time-matched control and five-day shade followed by five-day recovery groups [21]. Although its gene-level edgeR models completed, the supplied CSS annotation identifiers did not overlap the KEGG mapping resource, leaving zero mapped testable pathways in both contrasts. PRJNA915477 contained triplicate 60% black-net shade and triplicate non-shade ‘Zhongcha 102’ libraries. Twelve paired FASTQ files (19.2247 GB) were verified and quantified with kallisto 0.51.1 against the same Plant GARDEN CSS v2 coding sequences; all six quantifications were present, with pseudoalignment rates of 66.3–70.2%. The edgeR model retained 27,706 genes and identified 10,315 genes at FDR < 0.05; pathway analysis tested 127 KEGG pathways, 36 of which passed FDR < 0.05.

Each contributing BioProject was assigned equal study-level weight. Within CRA004002, the median normalized enrichment score across its five selected lower-light contrasts was calculated separately for every pathway; GSE97659, PRJNA1134925 and PRJNA915477 each contributed one selected lower-light score. Pathways available in at least three of the four BioProjects were retained, yielding 126 context pathways. Their median study-level score defined the external context vector. Each present zone-versus-CK pathway vector was compared with this vector over 119 overlapping pathways. Spearman rho and sign concordance were treated as descriptive summaries because KEGG pathways overlap and are not independent study replicates. Nominal *p* values and BH-adjusted values were retained in Supplementary Table S19 for transparency but were not used to claim independent validation. The main sensitivity check omitted each BioProject in turn. For the eight biologically selected pathways, recurrence was summarized by median score and majority sign fraction; low-powered sign-test results were reported only as supplementary context. The GSE142753 recovery comparison was recorded as not testable rather than interpreted as a null biological effect.

### 2.8. Reproducibility and Reporting

All scripts used fixed seed 20260728. Figure source tables, full significant-feature tables, pathway results, permutation summaries, sensitivity analyses, the public dataset qualification ledger and provenance notes are supplied in Supplementary Tables S1–S20. Analysis scripts, software environment files and logs are available from the corresponding author on reasonable request. Supplementary Table S21 compares the present archive with the related 2025 publication, and Supplementary Table S22 reports metabolite annotation levels, pooled-QC precision and pathway mapping limitations. Numeric audits confirmed plotted core values against source result files. Supplementary Table S23 lists all 15 analysis and raw sample identifiers and distinguishes confirmed replicate information from unavailable original field identifiers.

## 3. Results

### 3.1. Data Provenance and Quality Support Exploratory Field-Zone Comparisons but Not Installation-Level or Individual Physiology–Omics Comparisons

**Figure 1:**
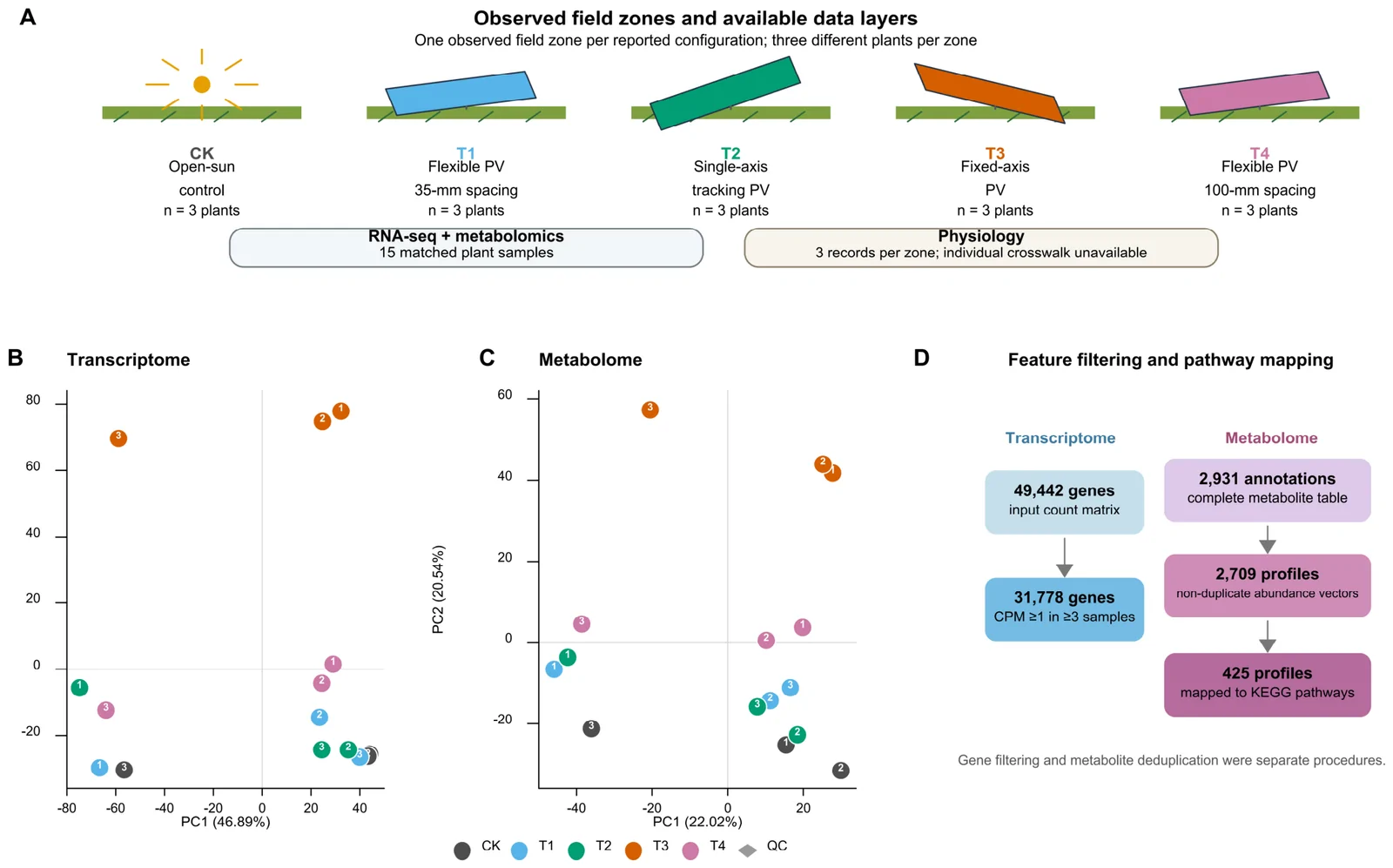
Study design and multi-omics quality control. (**A**) Open-sun control (CK) and four photovoltaic (PV) field zones (T1–T4), with three plant-level replicates per zone. Transcriptome and metabolome samples were matched; physiology records lacked an individual-level crosswalk. (**B**,**C**) Principal component analysis (PCA) of 31,778 genes and 2709 unique metabolite profiles, respectively; diamonds mark pooled quality control (QC) injections. (**D**) Data filtering and pathway mapping.

Fifteen biological samples were represented in both transcriptome and metabolome matrices, with three plant-level biological replicates assigned to each recorded zone, CK and T1–T4 (Figure 1A). The replicates in each zone came from different plants. Leaf material within a replicate was combined for sample preparation, but the three biological replicates were processed separately and were not pooled with one another. However, original plot maps, installation-level replication and randomization or blocking records were unavailable. The three biological replicates per zone therefore cannot be assumed to represent three independent photovoltaic installations. All results below describe differences among the observed field zones and samples; they do not identify a causal effect of panel type, tracking or shade dose.

The two differently branded physiology packages contained identical measurement workbooks and the same 15 assay codes and values. They were therefore counted as one assay source, not independent validation. The 15 physiology assay codes had no verified plant ID crosswalk to the omics labels; same-plant pairing remains unknown, and no individual-level trait–omics correlation was calculated.

After filtering, 31,778 of 49,442 genes were retained. Transcriptome principal components 1 and 2 explained 46.89% and 32.49% of the variance, and the mean within-zone pairwise Pearson correlation among sample gene-expression profiles was 0.952 (Figure 1B). Vendor QC tables recorded 47.8–60.0 million clean reads per library, Q30 values of 95.37–96.29%, and overall mapping rates of 88.11–89.74%. Metabolomics contained 2931 annotation rows with complete values for all 15 samples and three pooled QC injections; 2709 distinct exact abundance profiles entered the analysis. The median pooled-QC CV was 14.6%, and 86.9% of rows had CV ≤ 30%. Metabolome principal components 1 and 2 explained 22.02% and 20.54%, and the mean within-zone pairwise Pearson correlation among sample metabolite profiles was 0.932 (Figure 1C,D). Across both omics PCAs, T1-1, T2-1, T3-3, T4-3 and CK-3 fell on the negative side of PC1, while the other ten biological samples fell on the positive side. The available metadata contained no field block, processing batch or acquisition order variable that explained this shared structure. PC1 was therefore not interpreted solely as a field-zone effect.

### 3.2. Similar Total Chlorophyll Masks Broad Biochemical and Molecular Divergence Between T2 and T3

**Figure 2:**
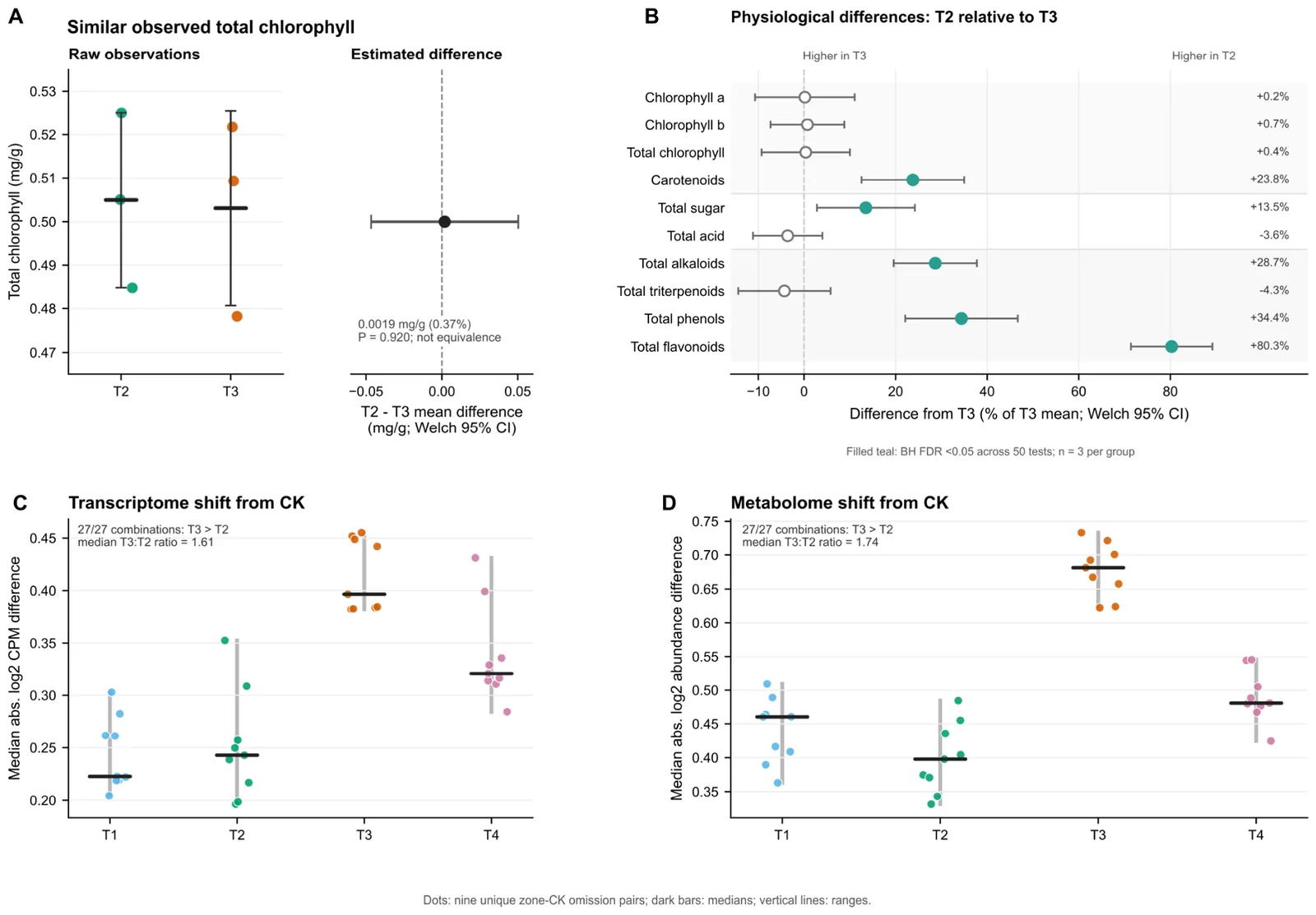
Physiological and molecular differences between T2 and T3. (**A**) Total chlorophyll observations and the T2–T3 mean difference with a Welch 95% confidence interval (CI); bars show means and standard deviations (SDs). (**B**) T2–T3 percent differences for ten traits with 95% CIs; filled points have Benjamini–Hochberg (BH) FDR < 0.05 across 50 tests. (**C**,**D**) Transcriptome and metabolome response magnitudes from the open-sun control (CK). Dots show nine unique omission pairs per contrast; bars show medians and ranges. T3 exceeded T2 in all 27 T2–T3–CK leave-one-per-zone combinations. In (**A**,**C**,**D**), blue, green, orange and pink identify T1, T2, T3 and T4, respectively; in (**B**), filled teal points indicate BH FDR < 0.05 and open grey points indicate BH FDR ≥ 0.05.

Mean total chlorophyll was 0.50495 mg g^−1^ in T2 and 0.50309 mg g^−1^ in T3, a difference of 0.00187 mg g^−1^ (0.37% relative to T3; Welch *p* = 0.920; Figure 2A). These observed group means are numerically similar but are not statistically equivalent because no biologically meaningful equivalence margin was specified. T2 also had higher mean carotenoids (+23.8%), total sugar (+13.5%), total alkaloids (+28.7%), total phenols (+34.4%) and total flavonoids (+80.3%) than T3 (Figure 2B). For these five traits, Welch *p* values had BH FDR < 0.05 across the defined 50-test physiological family. Each zone contained only three values, and the smallest attainable exact two-sided 3-versus-3 permutation *p* value is 0.1 (2 of 20 assignments), not an alternative significance threshold. These *p* values are exploratory sample-level summaries; raw observations, confidence intervals and effect sizes are more informative and do not support inference across independent photovoltaic installations.

The molecular response magnitudes differed even more strongly. At FDR < 0.05, T2 versus CK contained only 7 differentially expressed genes, compared with 6857 for T3 versus CK; 4946 genes differed directly between T2 and T3. The corresponding counts with an absolute log_2_ fold change ≥ 1 were 6, 4737 and 3659. For metabolites, 35 independent profiles differed in T2 versus CK and 523 in T3 versus CK, while 411 differed between T2 and T3. These counts describe the scale of statistical evidence but are affected by variance and power and are not interpreted as energetic “cost.”

We therefore computed a threshold-independent response index: the median absolute difference in group-mean log_2_ abundance across all retained features. Each individual zone–CK contrast had nine unique leave-one-zone/leave-one-CK pairs. For the primary T3:T2 comparison, one sample was omitted from each of T2, T3 and CK, yielding 3 × 3 × 3 = 27 possible omission combinations; T1 and T4 omissions were irrelevant because they did not change either response. T3 exceeded T2 in all 27 combinations for both transcriptome and metabolome response magnitudes (Figure 2C,D). The median T3:T2 response magnitude ratio was 1.61 (range 1.13–2.30) for transcript abundance and 1.74 (1.41–2.21) for metabolite abundance. Across all 243 all-zone combinations, T3 was the largest of the four CK contrasts in 92.6% of transcriptome combinations and all metabolome combinations. Thus, the broader T3 state shift was not driven by a single sample or a differential expression threshold.

### 3.3. Photosynthetic Carbon and Pigment Pathways Distinguish the Two Field Zones with Similar Chlorophyll Levels

**Figure 3:**
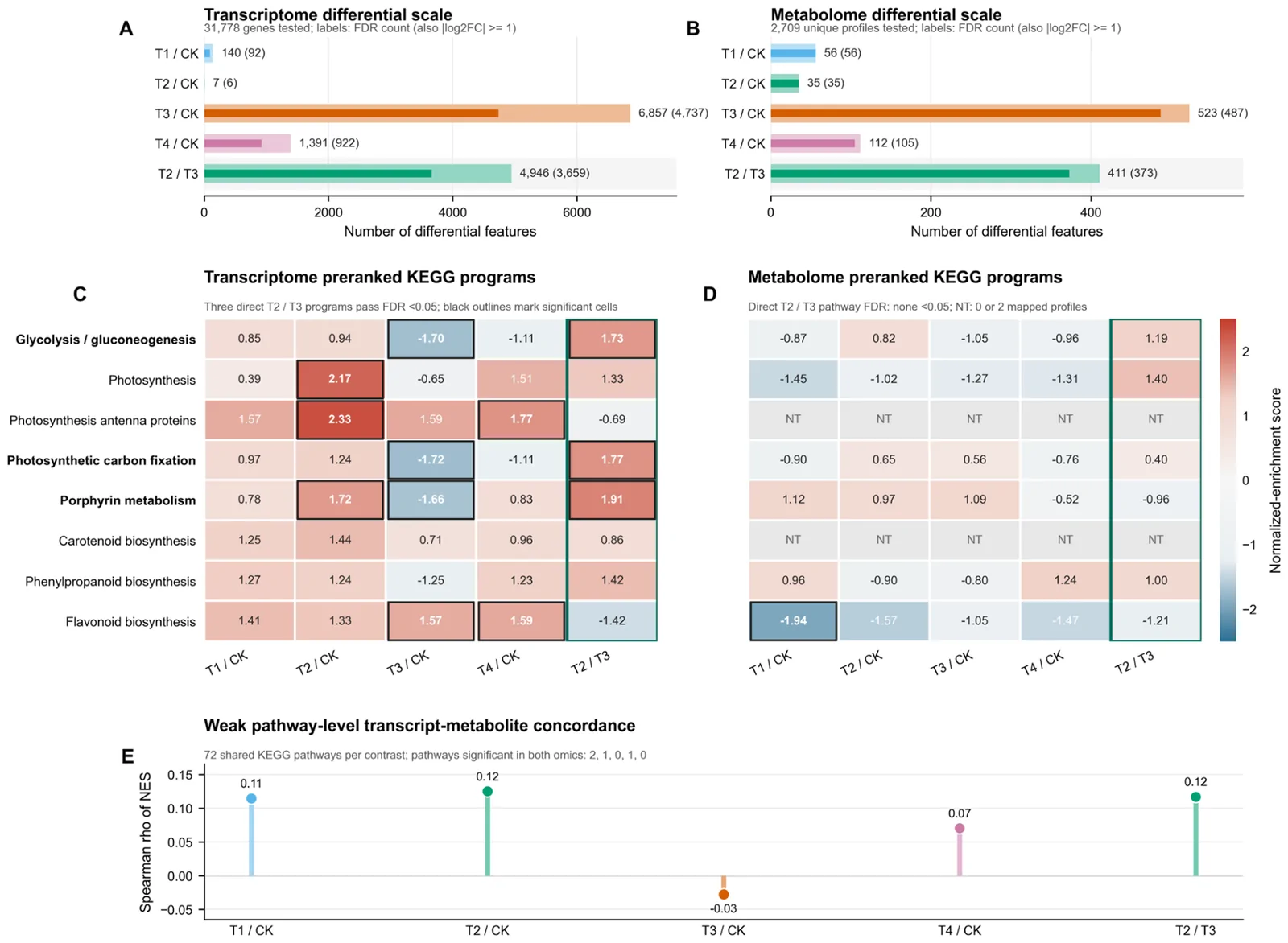
Differential features and pathway direction across field zones. (**A**,**B**) Counts of genes and independent metabolite profiles passing a false discovery rate (FDR) < 0.05; inset bars show the subset with an absolute log_2_ fold change ≥ 1. (**C**,**D**) Kyoto Encyclopedia of Genes and Genomes (KEGG) normalized enrichment scores for selected transcript and metabolite pathways. Outlines indicate FDR < 0.05, NT denotes not tested, and the green frame marks T2 versus T3. Positive scores favour the numerator zone. (**E**) Spearman correlations of transcript and metabolite pathway scores across 72 shared pathways. In (**A**,**B**,**E**), blue, green, orange and pink identify T1/CK, T2/CK, T3/CK and T4/CK, respectively; teal identifies T2/T3. In (**C**,**D**), red and blue indicate positive and negative enrichment scores, respectively, and grey indicates untested entries.

Preranked KEGG analysis resolved coherent differences not apparent from total chlorophyll (Figure 3C,D). Relative to CK, T2 showed positive enrichment of photosynthesis, photosynthesis antenna proteins and porphyrin metabolism (all FDR < 0.002). T3 showed negative enrichment of glycolysis/gluconeogenesis, carbon fixation in photosynthetic organisms and porphyrin metabolism. Direct T2-versus-T3 comparison placed glycolysis, carbon fixation and porphyrin metabolism toward T2. Within the observed dataset, the two recorded zones therefore had different pigment, light-harvesting and carbon metabolism states. These patterns do not identify the panel configuration or light variable that produced them.

Specialized metabolism showed a different form of divergence. Flavonoid biosynthesis transcripts were positively enriched in T3 versus CK, whereas total flavonoid concentration was markedly lower in T3 than T2. Across all shared KEGG pathways, transcript and metabolite normalized enrichment scores were only weakly correlated (Spearman ρ from −0.028 to 0.125, depending on contrast; Figure 3E), and T3 had no pathway significant at FDR < 0.05 in both omics. The transcript and accumulated metabolite signals were therefore not concordant, and the present data do not establish a direct relationship between flavonoid pathway transcription and total flavonoid abundance.

### 3.4. Exact Permutations Identify Selective Rather than Global Cross-Omics Coupling

**Figure 4:**
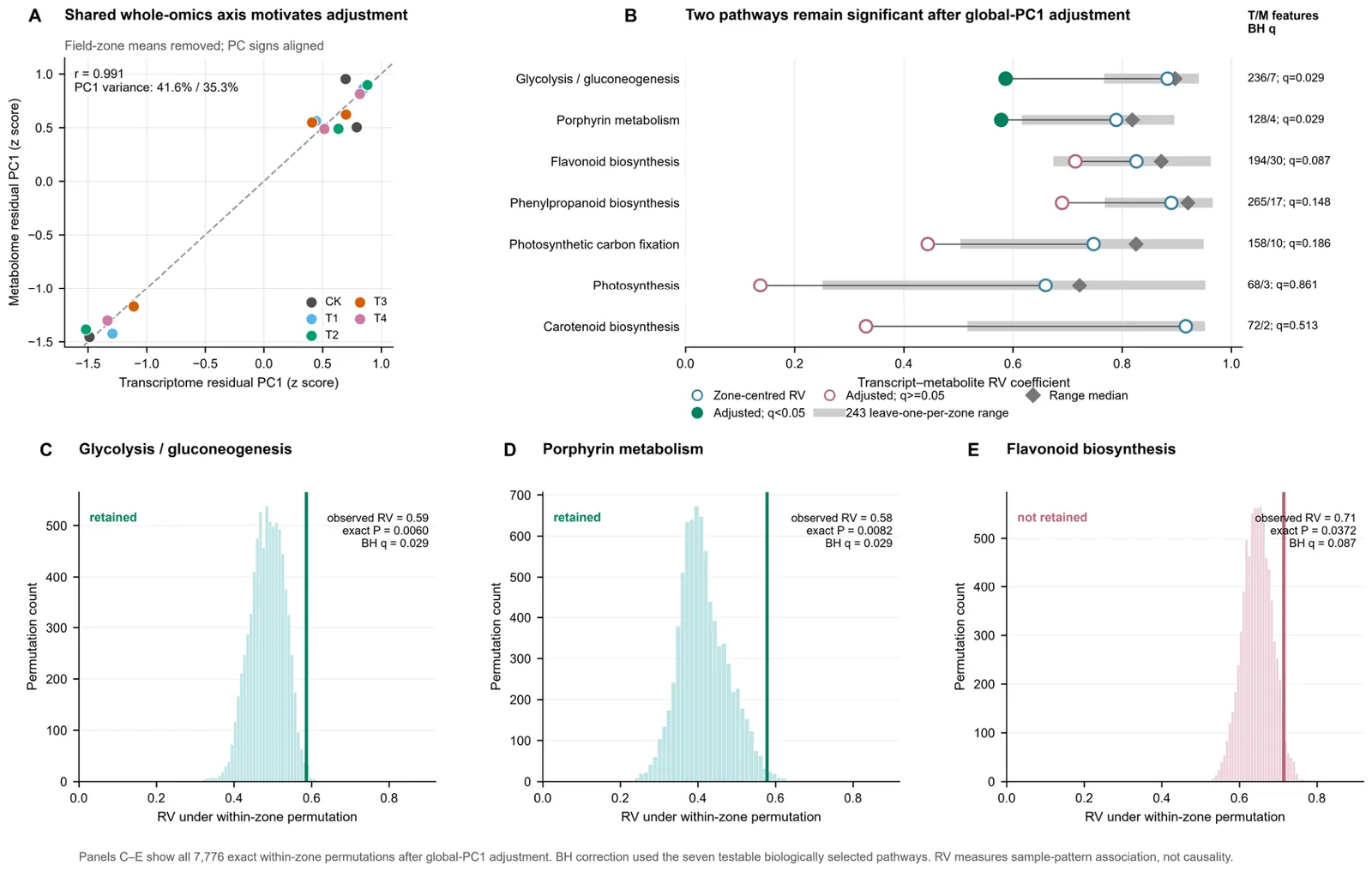
Selective cross-omics sample-pattern coupling. (**A**) Zone-centred whole-omics first principal component (PC1) scores for 15 samples; their correlation is descriptive. (**B**) Global PC1-adjusted RV sample-pattern association coefficients for seven testable pathways. Diamonds and grey bars show medians and ranges across 243 leave-one-per-zone combinations; labels show feature counts and Benjamini–Hochberg (BH)-adjusted *q* values. (**C**–**E**) Exact permutation null distributions for glycolysis/gluconeogenesis, porphyrin metabolism and flavonoid biosynthesis; vertical lines mark observed RV coefficients.

Of the 2709 independent metabolite profiles, 425 had KEGG pathway mappings. Seven of the eight biologically selected pathways contained mapped features in both modalities; photosynthesis antenna proteins lacked an independent mapped metabolite profile and could not be tested (Figure 4).

All seven testable pathways passed FDR correction before global-factor adjustment. However, the transcriptome and metabolome had a strongly aligned dominant within-zone whole-omics axis (Pearson *r* = 0.991 after PC-sign alignment; Figure 4A), indicating that broad individual or handling variation could inflate pathway-level agreement. After removing each modality’s leading whole-omics within-zone PC1 and recomputing the tests, only glycolysis/gluconeogenesis (adjusted RV = 0.586, exact *p* = 0.00604, BH *q* = 0.0288) and porphyrin metabolism (adjusted RV = 0.578, exact *p* = 0.00823, BH *q* = 0.0288) remained significant (Figure 4B–D). Flavonoid biosynthesis had a nominal exact *p* = 0.0372 but did not pass BH correction (*q* = 0.0867; Figure 4E), and carotenoid biosynthesis contained only two mapped independent profiles. The supported conclusion is selective sample-pattern coupling in glycolysis and porphyrin metabolism, not a dense causal regulatory network.

### 3.5. Public Tea Datasets Provide Cross-Study Context for the Recorded Field-Zone Programmes

**Figure 5:**
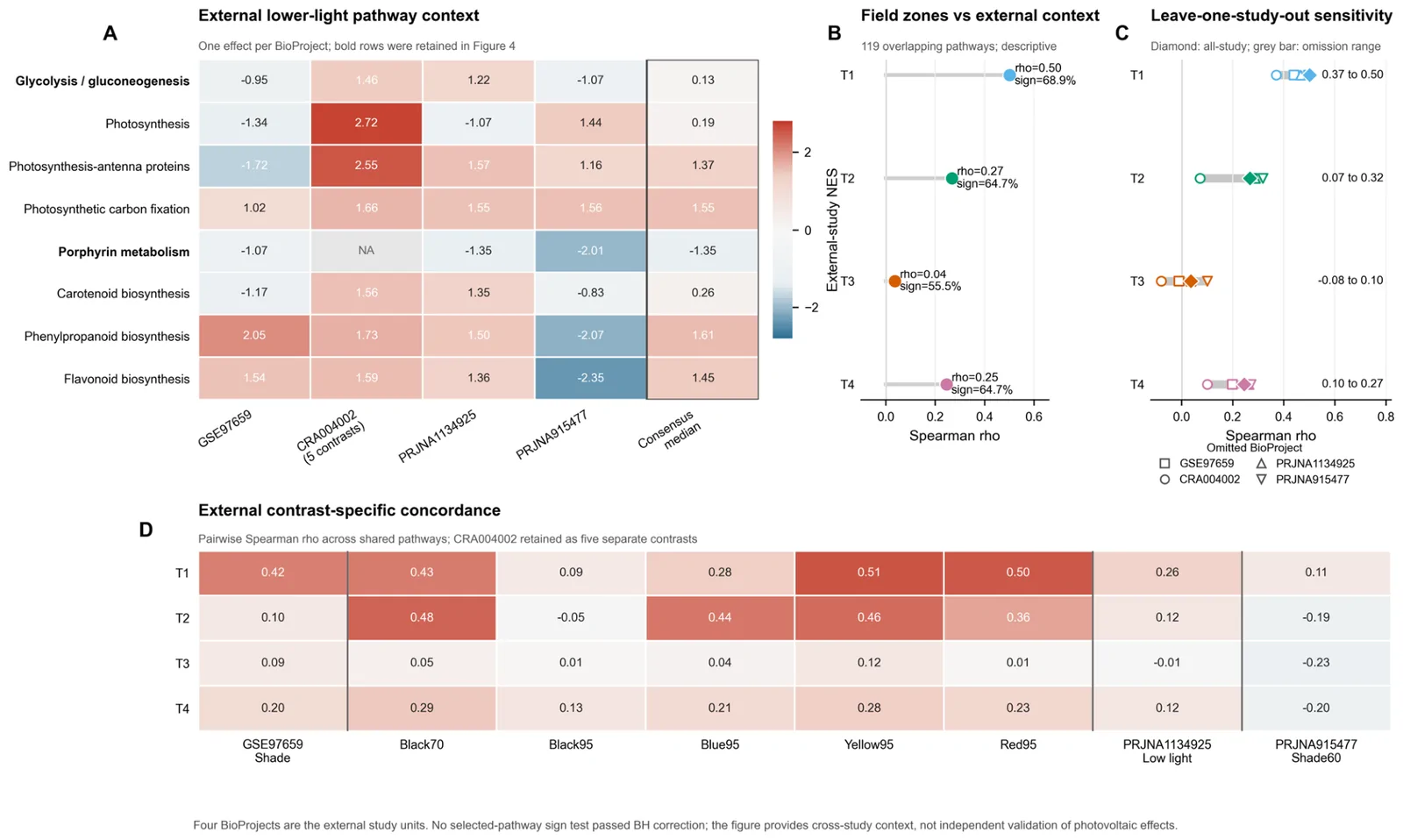
Cross-study pathway contextualization. (**A**) Pathway enrichment across four public tea BioProjects and their median context vector; grey denotes unavailable estimates and bold denotes pathways retained in Figure 4. (**B**) Spearman rank concordance between field zone and external context profiles. (**C**) Leave-one-study-out sensitivity; diamonds show all-study estimates, shapes identify omitted studies, and grey bars show omission ranges. (**D**) Concordance with individual lower-light contrasts. Positive external scores indicate higher abundance under lower light.

To place the field-zone responses in a broader lower-light context, we compared the present study pathway profiles with independently processed public tea transcriptomes (Figure 5). The comparison showed the clearest and most stable concordance for T1, whereas the other field zones showed weaker or study-dependent agreement. Among the cross-omically supported pathways, porphyrin metabolism showed a more consistent direction than glycolysis/gluconeogenesis across the external studies. However, no pathway-level comparison remained significant after multiple-testing correction. These results provide descriptive cross-study context rather than independent validation of field-zone or photovoltaic configuration effects. Dataset eligibility, contrasts, processing, exclusions and complete sensitivity results are provided in the Supplementary Methods and Results and Supplementary Tables S19 and S20.

## 4. Discussion

The observed T2 and T3 zones had nearly identical mean total chlorophyll but large differences in biochemical composition, molecular response magnitude and pathway state. Chlorophyll accumulation alone therefore did not identify a common molecular profile within this dataset. The result describes field-zone-associated molecular variation within an agrivoltaic setting. Because each photovoltaic configuration occurred in only one zone and the study lacked independent installation replicates and time-resolved exposure measurements, panel geometry or operation cannot be separated from zone position or other field differences.

The contrast between T2 and T3 was not driven only by differential feature counts. Continuous response magnitudes consistently placed T3 farther from the sunlight control in every transcriptome and metabolome leave-one-per-zone resample. The pathway results localized part of this divergence: T2 maintained positive photosynthetic and porphyrin programmes, whereas T3 showed negative carbon fixation, glycolytic and porphyrin enrichment. Nevertheless, terms such as “efficiency,” “stress cost” or “superior performance” would be premature. The dataset lacks photosynthetic gas exchange, biomass, yield, energy production and resource-use measurements needed to connect molecular displacement to agronomic benefit or fitness.

The cross-omics results also argue for restraint. A naïve analysis suggested widespread transcript–metabolite coupling, but most signals weakened after removal of a shared whole-omics latent factor. Only glycolysis/gluconeogenesis and porphyrin metabolism survived this sensitivity analysis. The RV coefficients describe sample-pattern similarity rather than directionality or molecular causation, so these pathways remain focused hypotheses for validation using photosynthetic, carbohydrate flux, chlorophyll intermediate and enzyme activity measurements.

Previous tea studies support both pathways as light responsive but also show dependence on exposure regime and cultivar. Long-term shading suppressed glycolysis and related sugar metabolism pathways in tea leaves and roots [22], consistent with the negative glycolysis/gluconeogenesis enrichment in T3 and T4 relative to CK, but not with the positive T2 response. Shade treatment of the light-sensitive cultivar ‘Baijiguan’ strongly affected porphyrin and chlorophyll metabolism [23], while a high-light multi-omics study identified light-associated regulation of *CsPORC* [20]. Thus, the implication of tetrapyrrole/chlorophyll metabolism is consistent with previous work, whereas the opposing porphyrin metabolism responses in T2 and T3 were observed in these field zones; whether they are specific cannot be determined from the available comparisons. Differences in cultivar, exposure history and microclimate preclude attributing these contrasts to photovoltaic configuration alone.

Flavonoid metabolism illustrates why transcript and accumulated metabolite abundance cannot be treated as interchangeable. T3 showed positive flavonoid transcript enrichment but lower total flavonoids than T2, and the pathway did not survive adjusted cross-omics testing. Tea shade responses are strongly dependent on cultivar, spectrum, duration and tissue [4,5,6,7,8,9]. Compensation, altered branch allocation, transport or delayed accumulation could all produce opposing layer-level signals. Time-resolved sampling and targeted metabolite validation would be needed to distinguish these explanations.

Cross-study comparisons provided context without converting association into mechanism. T1 had the largest and most stable descriptive rank concordance with the four-BioProject lower-light vector. T2 and T4 were weaker and more sensitive to BioProject omission, whereas T3 remained near zero. This pattern may reflect local microclimate, cultivar, field position or another unmeasured factor; it is not evidence of a fixed-PV mechanism. The absence of original plot maps, independent installation replicates, sample-to-plant identifier crosswalks and time-resolved environmental measurements prevents causal interpretation of configuration-specific effects.

The related 2025 study assessed chlorophyll fluorescence and yield in the same CK/T1–T4 zones [10], and Ruifang Wang and Jie Liu are authors of both papers. All six current authors signed a declaration that the present study used a separate sampling batch and did not reuse biological samples or datasets. Row-level chain-of-custody records remain incomplete (Section 2.1; Supplementary Materials), so the no-reuse statement is author confirmed, not an independent sample-by-sample audit. This study is a molecular extension of the same field programme, not independent field validation.

The study has five major limitations. First, independent photovoltaic installations, field blocks and plot-level replication were not documented; the three plant-level biological replicates per zone cannot be assumed to be installation-level replicates. Second, n = 3 per zone limits precision and makes exact physiology tests coarse. Third, the physiology assay-to-omics crosswalk is unresolved, preventing individual-level trait–omics integration. Fourth, only 15.7% of independent metabolite profiles mapped to KEGG, and annotations were database matches without documented authentic-standard confirmation. Fifth, field exposure information was limited to summary lux values and shading estimates; time-resolved PPFD, spectral composition, microclimate measurements and functional validation were unavailable. The results therefore describe exploratory molecular differences among recorded field zones. Future studies should replicate each photovoltaic configuration across independent plots and pair configuration-resolved light and microclimate measurements with diurnal physiology, yield, quality chemistry and targeted molecular validation.

## 5. Conclusions

Similar observed total chlorophyll levels in T2 and T3 coexisted with markedly different biochemical, transcriptomic and metabolomic states, and T3 showed greater molecular displacement from CK than T2 across all leave-one-per-zone resamples in both omics layers. After adjustment for the dominant whole-omics factor, sample-pattern coupling remained supported only for glycolysis/gluconeogenesis and porphyrin metabolism. Comparisons with public tea datasets provided context but not independent validation. Because each photovoltaic configuration occurred in only one recorded field zone and independent installation replicates and time-resolved exposure measurements were unavailable, these findings describe field-zone-associated molecular variation rather than causal effects of photovoltaic configurations. Replicated experiments with configuration-resolved light, microclimate and agronomic measurements are needed to test mechanism and practical consequences.

## Data Availability

The public datasets used for cross-study contextualization are available under GSE97659/PRJNA356134, CRA004002/PRJCA004626, PRJNA1134925, GSE142753/PRJNA598211 and PRJNA915477. GSE142753 was not usable for pathway-level comparison; the other four BioProjects contributed to the context vector. The complete public data screening ledger and source links are provided in Supplementary Table S20. The study RNA-seq data are deposited in the Genome Sequence Archive under BioProject PRJCA065311 and are under controlled release, with public release scheduled for 27 May 2028. During the pre-release period, the RNA-seq data and the underlying metabolomics, physiology, processed matrices, analysis scripts, software environment files and figure-source tables are available from the corresponding author on reasonable request. Processed results and the public data screening ledger are included in the Supplementary Tables.

## Supplementary Materials

The following supporting information can be downloaded at: https://www.mdpi.com/article/10.3390/genes17101221/s1.
