# Supplementary material for: Field-Zone-Associated Transcriptomic and Metabolic Divergence Despite Nearly Identical Observed Chlorophyll in an Agrivoltaic Tea Setting
Source: Genes (Basel). 2026 Oct 1;17(10):1221. doi: 10.3390/genes17101221 (PMC13704113; doi:10.3390/genes17101221)
Supplement: Supplementary file 1 [file genes-17-01221-s001.zip › 03_Supplementary_Methods_and_Results.pdf]

Supplementary Methods and Results

Supplementary Methods

Source provenance and immutable inputs

The original vendor archive was inventoried without modification. Core inputs were copied into paper_project/source_data and checksummed in paper_project/data_manifest. Two physiology delivery packages (JWBX and RW) were compared at OOXML cell/formula/cached-value level. Although their binary hashes and administrative report branding differed, all populated workbook values were identical; they were treated as one assay chain.

Samples and design restrictions

Transcriptome, metabolome and vendor joint-analysis matrices contained the same 15 analysis labels: CK and T1–T4, with three plant-level biological replicates assigned to each recorded field zone. The replicates in each zone came from three different plants. Leaf material within each plant-level replicate was combined for sample preparation, but the three biological replicates were processed separately and were not pooled with one another. A derived background table mapped the analysis labels to raw sample IDs A24213775c–A24213789c. The archive did not include independent photovoltaic-installation IDs, field blocks, plot IDs or the plant identifier linked to each analysis label. Project information identifies 'Yunhuang No. 1' scions on 19-year-old 'Yunkang 10' rootstocks. Zone membership was retained. Physiology reports placed three project IDs in a merged group cell beside three assay-code rows; because no original one-to-one crosswalk was supplied, physiology was analysed at zone level and no individual trait–omics correlation was performed. Supplementary Table S23 separates confirmed sample-design information from the original row-level identifiers that remain unavailable.

RNA-seq matrix and quality control

The discovery count matrix contained 49,442 unique genes, nonnegative integer counts and no missing values. Genes with CPM ≥1 in at least three samples were retained (31,778). TMM-normalized $\log_2$ CPM values were used for PCA and sample correlations. The project-specific vendor report recorded fastp 0.23.2 for read filtering, HISAT2 2.2.1 alignment to GWHAZTZ00000000, StringTie 2.1.6 transcript assembly and featureCounts 2.0.3 gene quantification. It also reported 47.8–60.0 million clean reads per library, Q30 of 95.37–96.29%, overall mapping of 88.11–89.74% and uniquely mapped reads of 83.04–84.81%.

Metabolomics matrix and quality control

The raw vendor workbook contained 2,932 annotation rows. Of these, 2,931 had complete values for the 15 study samples and three pooled QC injections and formed the primary matrix. The vendor assigned 802, 493 and 1,637 rows to match levels 1, 2 and 3. These are vendor database-match categories, not authentic-standard MSI identification levels. Across the 2,931

complete rows, the pooled-QC median CV was 14.61%; 1,971 rows (67.25%) had CV≤20%, 2,547 (86.90%) had CV≤30%, and all had CV<50%. Exact equality across all 18 columns identified 2,709 independent profiles. Primary inferential analyses retained one abundance vector per exact profile.

The vendor report documented vacuum freeze-drying for 63 h and grinding at 30 Hz for 1.5 min. A 50-mg powder aliquot was extracted with 1,200 µL prechilled 70% methanol containing a vendor-reported internal standard at 250 µg/mL, prepared from a 1,000 µg/mL stock. The internal-standard compound was not identified. The extract underwent six vortex cycles, centrifugation at 12,000 rpm for 3 min and 0.22-µm filtration. UPLC used an ExionLC AD and Agilent SB-C18 column (1.8 µm, 2.1×100 mm), with water/acetonitrile containing 0.1% formic acid, a 5–95% B gradient over 9 min, 0.35 mL min−1, 40 °C and 2-µL injection. Triple-quadrupole MRM data were processed in Analyst 1.6.3 and MultiQuant. The exact mass-spectrometer model, raw spectra, authentic-standard confirmation, acquisition order, sample randomization and a separate signal-drift correction procedure were not supplied.

Physiology statistics

Ten traits were extracted with three assay rows per recorded zone. Zone means, SDs, T1–CK through T4–CK and T2–T3 Welch contrasts, Hedges' g and 95% confidence intervals were calculated. All 20 label assignments were enumerated for exact two-sided 3-versus-3 permutation context. Its smallest attainable *p* value is 0.1 (2 of 20 assignments), a resolution limit rather than an alternative significance threshold. Welch *p* values from the 10 traits × five zone contrasts were BH-corrected together as one family of 50 tests; this was prioritized for reporting in the retrospective reanalysis, with FDR<0.05 as the threshold. BH FDR within each trait across five contrasts was also reported. No equivalence test was performed because an equivalence margin had not been specified before analysis. Because independent field installations were not documented, *p* values are exploratory summaries of the supplied sample values and do not support installation-level inference.

Differential analysis and pathway enrichment

RNA counts were fitted with a robust edgeR quasi-likelihood five-zone no-intercept model after TMM normalization. Metabolite profiles were fitted with limma robust/trend moderation under the same design. Contrasts were T1–CK, T2–CK, T3–CK, T4–CK and T2–T3. Benjamini–Hochberg FDR<0.05 defined model-based statistical significance; an absolute $\log_2$ fold change ≥ 1 was a secondary effect-size description, not an extra significance rule. These FDR values describe contrasts among the supplied samples and do not estimate uncertainty across independent photovoltaic installations.

Transcript KEGG enrichment used signed square-root quasi-likelihood F statistics, fgsea pathways of 10–500 genes and BH correction. Of 2,709 independent metabolite profiles, 425 (15.7%) had KEGG pathway mappings. All annotation-to-pathway links were carried to the corresponding exact-profile ID. A profile could enter more than one supported pathway when alternative annotations linked it to different pathways, but it was counted only once within any one pathway. Normalization sensitivity compared alternative sample scaling and centring rules and recorded rank and direction concordance.

Threshold-independent response magnitude

For each zone-versus-CK contrast, response magnitude was the median absolute difference between zone and CK group means over all 31,778 retained genes or 2,709 unique exact metabolite profiles. The full five-zone enumeration contained 3^5=243 omission combinations and was retained for ranking all four zone–CK contrasts. Each single zone–CK distribution contains nine unique omission pairs. For the primary T3:T2 comparison, T1 and T4 omissions do not change either value, leaving 3^3=27 unique T2–T3–CK combinations.

Group-aware cross-omics exact permutation

Eight KEGG pathways were biologically selected because they represented the pigment, photosynthesis, carbon and specialized-metabolism processes central to the study question: glycolysis/gluconeogenesis, photosynthesis, photosynthesis-antenna proteins, carbon fixation in photosynthetic organisms, porphyrin metabolism, carotenoid biosynthesis, phenylpropanoid biosynthesis and flavonoid biosynthesis. No prospectively registered pathway protocol was available; these analyses are exploratory rather than prespecified. Seven pathways had enough mapped features in both modalities to be tested. Within each testable pathway, transcript and metabolite features were standardized after zone means were removed. The RV coefficient between transcript and metabolite 15×15 sample-similarity matrices was tested against all 6^5=7,776 metabolite-label permutations within zone. Exact p values were BH-corrected across the seven testable pathways. Sensitivity analysis removed each modality's dominant within-zone whole-omics PC1 before recomputation, and the adjusted exact p values were separately BH-corrected across the same seven pathways. All 243 leave-one-per-zone combinations were also evaluated for cross-omics sensitivity.

Public-dataset discovery and cross-study contextualization

On 28 July 2026, NCBI SRA was queried through E-utilities for *Camellia sinensis* shade, shading and light studies. All 23 returned BioProjects were reviewed before downstream expression direction was examined. Qualification decisions and exclusions were fixed in Supplementary Table S20. Four BioProjects contributed to the final lower-light context vector: GSE97659/PRJNA356134, CRA004002/PRJCA004626, PRJNA1134925 and PRJNA915477. Each contributed one study-level NES per pathway; CRA004002 was collapsed by the median of five selected shade or filter contrasts. Pathways present in at least three studies were retained. Spearman rho and sign concordance between each present zone-versus-CK pathway vector and the external vector were treated as descriptive summaries because the 119 overlapping KEGG pathways are not independent study replicates. Nominal p values and BH-adjusted values were retained in Supplementary Table S19 only for transparency. Leave-one-BioProject-out analysis generated four omissions per zone and provided the main sensitivity check. This analysis is cross-study contextualization, not independent validation of a photovoltaic configuration or mechanism.

PRJNA915477 consisted of six paired-end runs (12 FASTQ, 19.2247 GB). All files were byte/metadata verified and quantified with kallisto 0.51.1 against Plant GARDEN CSS v2 CDS. Pseudoalignment rates ranged from 66.3% to 70.2%. The edgeR and KEGG analyses completed on the designated server.

Software and reproducibility

Primary server versions were R 4.4.3, edgeR 4.4.0, limma 3.62.1, fgsea 1.32.2 and kallisto 0.51.1. The fixed seed was 20260728. Scripts, Slurm wrappers, compact outputs, source tables, metadata and logs are available from the corresponding author on reasonable request.

Supplementary Results

Provenance and data integrity

The 15 sample labels matched across transcriptome and metabolome matrices. Physiology workbooks were cell-for-cell duplicates and were counted once. Project information defines three plant-level biological replicates from different plants in each zone and states that replicates were processed separately without pooling across replicates. The discovery archive did not contain raw RNA FASTQ, raw metabolomics instrument files, an original field map, independent installation records, row-level plant identifiers or a verified individual physiology–omics crosswalk.

Omics QC

Transcriptome PCA explained 46.89% and 32.49% on PC1 and PC2. Mean within-zone pairwise Pearson correlation among sample gene-expression profiles was 0.9520 (minimum 0.9188); the library-count-sum CV was 6.86%. Metabolome PCA explained 22.02% and 20.54%; mean within-zone pairwise Pearson correlation among sample metabolite profiles was 0.9323 (minimum 0.9022). Transcriptome and metabolome whole-omics sample scores were strongly correlated, including zone-centred PC1 r=0.9910, which motivated the global-factor sensitivity analysis.

Chlorophyll and biochemical traits

T2 and T3 mean total chlorophyll were 0.50495 and 0.50309 mg g^{-1}, respectively (Welch p=0.920). This supports the phrase numerically similar but does not establish statistical equivalence. Carotenoids, total sugar, total alkaloids, total phenols and total flavonoids had larger T2–T3 effect estimates and had BH FDR<0.05 across the defined 50-test physiological family. Because n=3 per group and the exact permutation limit is p=0.1, the raw values, intervals and effect sizes remain more informative than a binary significance label.

Differential scale and response magnitude

At FDR<0.05, T2–CK and T3–CK contained 7 and 6,857 differentially expressed genes, and 35 and 523 differential unique metabolite profiles. Direct T2–T3 comparisons contained 4,946 genes and 411 metabolite profiles. T3 response magnitude exceeded T2 in 27/27 unique T2–T3–CK combinations in both modalities. Median T3:T2 ratios were 1.608 for the transcriptome and 1.740 for the metabolome. The full 243 five-zone combinations were retained only for ranking all four zone–CK contrasts and for audit. These values summarize the supplied samples and are not estimates across independently replicated photovoltaic installations.

Pathway direction and cross-omics sensitivity

T2 showed positive photosynthesis, antenna-protein and porphyrin transcript signatures relative to CK, whereas T3 showed negative glycolysis/gluconeogenesis, carbon fixation and porphyrin signatures. Seven pathways were testable. All passed the unadjusted exact cross-omics analysis after BH correction across those seven pathways, but only glycolysis/gluconeogenesis (adjusted RV=0.586, exact p=0.00604, BH FDR across seven pathways=0.0288) and porphyrin metabolism (adjusted RV=0.578, exact p=0.00823, BH FDR=0.0288) survived removal of the dominant global PC1. These are sample-pattern associations, not causal gene–metabolite networks.

Public-dataset qualification and lower-light context

Of 23 screened BioProjects, 12 were excluded with recorded reasons; qualified and provisional projects were expanded to project-complete SRA metadata. Four BioProjects contributed one study-level effect each to the final context vector. A total of 126 pathways were available in at least three studies; 119 overlapped local pathway tests. The 119 pathways are overlapping biological features, not 119 independent study replicates.

T1 had the strongest descriptive correlation with the external context vector (Spearman rho=0.502; sign concordance 68.9%), followed by T2 (rho=0.268; 64.7%) and T4 (rho=0.246; 64.7%). T3 was near zero (rho=0.037; 55.5%). Leave-one-BioProject-out rho ranges were 0.37–0.50 for T1, 0.07–0.32 for T2, 0.10–0.27 for T4 and −0.08–0.10 for T3. Nominal p values and BH FDR remain in Supplementary Table S19 as descriptive calculations only. No individual biologically selected pathway passed pathway-specific sign-test FDR. This is cross-study contextualization, not independent validation of a pathway, field zone or photovoltaic configuration. Across the eight individual external contrasts shown in Figure 5D, T1 correlations were positive in 8/8 contrasts (ρ=0.086–0.506), T4 correlations were positive in 7/8 (ρ=−0.195–0.290), T2 was heterogeneous (ρ=−0.192–0.476), and T3 was weak or negative (ρ=−0.233–0.115). These contrast-level values are descriptive and are listed in Supplementary Table S19.

GSE142753 produced gene-level edgeR results but zero testable mapped KEGG pathways in both the shade and recovery contrasts. The intended recovery comparison was therefore not testable and was not reclassified as a biological null result.

Limitations and alternative explanations

Alternative explanations include field position and unmeasured exposures rather than photovoltaic hardware. The three biological replicates per zone came from different plants, not independent PV installations; configuration and zone are confounded. Small n, incomplete metabolite identification and no functional validation limit inference to association. A related 2025 Pu'er study used the same CK/T1–T4 field labels and relative light descriptors, the same early-June/25-day schedule and third-functional-leaf sampling, and reported a TES-1334A protocol at 13:00–14:00. Ruifang Wang and Jie Liu are authors of both papers. All six authors signed a declaration that this work used a separate sampling batch and reused no biological samples or datasets, but original row-level chain-of-custody records were unavailable for independent audit. The present work is a molecular extension of the same field programme, not independent validation. Supplementary Table S21 compares the studies; Table S23 distinguishes confirmed replicate information from missing field identifiers.
